# Effects of Temperature on the Development and Population Growth of the Sycamore Lace Bug, *Corythucha ciliata*

**DOI:** 10.1673/031.011.0116

**Published:** 2011-02-14

**Authors:** Rui-Ting Ju, Feng Wang, Bo Li

**Affiliations:** ^1^ Coastal Ecosystems Research Station of the Yangtze River Estuary, Ministry of Education Key Laboratory for Biodiversity Science and Ecological Engineering, Institute of Biodiversity Science, Fudan University, Shanghai 200433, China; ^2^ Shanghai Institute of Landscape Gardening Science, Shanghai 200232, China

**Keywords:** fecundity, life table, thermal constant, threshold temperature

## Abstract

The sycamore lace bug, *Corythucha ciliata* (Say) (Hemiptera: Tingidae), is an important invasive exotic pest of *Platanus* (Proteales: Platanaceae) trees in China. The objective of this study was to determine the effects of temperature on *C. ciliata* in the laboratory so that forecasting models based on heat accumulation units could be developed for the pest. Development and fecundity of *C. ciliata* reared on leaves of London plane tree (*Platanus* × *acerifolia*) were investigated at seven constant temperatures (16, 19, 22, 26, 30, 33, and 36° C) and at a relative humidity of 80% with a photoperiod of 14:10 (L:D). The developmental time was found to significantly decrease with increasing temperature. The developmental time from egg hatching to adult emergence was respectively 47.6, 35.0, 24.1, 20.0, and 17.1 days at the temperatures of 19, 22, 26, 30, and 33° C. *C. ciliata* could not complete full development at 16° and 36° C. The developmental threshold temperature (C) estimated for egg-to-adult was 11.17° C, with a thermal constant of (*K*) 370.57 degree-days. Longevity of females was found to be the shortest, 17.7 days at 33° C and the longest, 58.9 days at 16° C, and that of males was the shortest, 19.7 days at 33° C and the longest, 59.7 days at 16° C. Fecundity was the highest at 30° C, being 286.8 eggs per female over an oviposition period of 8.9 days. Female lifetime fecundity was reduced at other temperatures, being the lowest (87.7 eggs per female) at 19° C. The population trend index (*I*) of *C. ciliata* was the highest (130.1) at 30° C and the lowest (24.9) at 19° C. Therefore, the optimal developmental temperature for *C. ciliata* was determined to be 30° C.

## Introduction

Insects are poikilothermic animals that are largely affected by various environmental factors. Among all the climatic factors, temperature has probably the greatest effect on insect development (Taylor 1981; Pedigo 1989). Previous studies have shown that temperature influences various biological characteristics of insects such as sex-ratio (Zheng et al. 2008), adult life-span, survival, fecundity, and fertility (Yang et al. 1994; Dreyer and Baumagartner 1996; Infante 2000). As a result temperature profoundly affects colonization, distribution, abundance, behavior, life history, and fitness of insects (Cossins and Bowler 1987; Denlinger and Yocum 1998; James et al. 2002; Hoffmann et al. 2003). Therefore, information on the thermal requirements of invasive insect pest development has important implications for control programs as temperature determines the population growth and size of invasive pests and their variation under different conditions (Kang et al. 2009).

The sycamore lace bug, *Corythucha ciliata* (Say) (Hemiptera: Tingidae), is a relatively new invasive exotic pest infesting *Platanus* (Proteales: Platanaceae) trees in China. It was first found in China in 2002 (Streito, 2006), and spread rapidly to 11 provinces over a period of less than a decade (Li et al. 2007; Ju et al. 2009; Ju and Li 2010). *Corythucha ciliata* originated in North America (Halbert and Meeker 1998), and is now widely distributed in Europe and eastern Asia (Maceljski 1986; Chung et al. 1996; Tokihiro et al. 2003). Both adults and nymphs of *C. ciliata* feed on the underside of leaves and produce small chlorotic stippling on the upper leaf surface. Leaf undersides appear characteristically black or dark brown varnish-spotted due to lace bug excrement. Their injury reduces photosynthesis and respiration of host plants and also affects aesthetical value of the trees. As a result foliage becomes bronzed and leaves may fall earlier, in late summer (Halbert and Meeker 1998). Several-years of severe damage by *C. ciliata,* combined with the effects of other environmental factors, may kill the trees (Barnard and Dixon 1983). Management recommendations include repeated applications of organophosphorous, synthetic pyrethroid, imidacloprid, thiamethoxam, or acetamiprid insecticides to avoid significant damage (Kim et al. 2000; Ju et al. 2009).

Adults of *C. ciliata* overwinter under the exfoliation of the outer bark of the host tree or in other protected places. Its eggs are inserted into the lower leaf mesophyll. Five immature instars are observed and five generations per year can be completed in Wuhan region, China (Xia et al. 2007). As a new invasive exotic pest, *C. ciliata* has not well been studied in relation to invasion biology in its non-native range in China. In Korea, previous studies have examined its thermal biology (Kim et al. 1999; Song and Cho 2000). However, in China, these data are so limited that they are inadequate to predict population development because geographical differences in response to temperature often result in differences in threshold temperatures and thermal unit requirements for development, and these differences further serve to synchronize species having a broad geographic distribution with local climatic conditions (Braman and Pendley 1993).

In recent years there has been an increasing interest in finding novel, powerful, target-selective, and environment-friendly pesticides to control *C. ciliata* (Ju et al. 2009). There are more studies on *Corythucha* sp. (Braman and Pendley 1993; Rédei et al. 2004; Bernardinelli 2006) and some other Tingidae species, like *Stephanitis* sp. (Braman et al. 1992; Shrewsbury and Raupp 2000; Klingeman et al. 2001; Stewart et al. 2002; Aysal and Kivan 2008), in the USA and Eastern Europe. Forecasting models based on heat accumulation units should be developed for *C. ciliata* like those reported for the pear lace bug, *Stephanitis pyri* (Aysal and Kıvan 2008). Such models for predicting insect development are important for insect control, which can improve the timing of pesticide application with minimal use of pesticides (Ascerno 1991). The objectives of this study were to determine the effects of temperature on the developmental periods, fecundity, adult longevity, and survivorship of *C. ciliata* under controlled environmental conditions, and to determine the thermal constant (*K*) and the lower developmental threshold (C) for *C. ciliata.* Understanding the optimal temperatures for the major phenologically-related parameters of *C. ciliata* (e.g., development, fecundity, and longevity) would be helpful to the efficient control of this species in invaded areas as addressed in other species (e.g., Zheng et al. 2008). Such information can also be of use to predicting the potential range of *C. ciliata* in China, where *Platanus* trees are widely planted in urban areas.

## Materials and Methods

### Laboratory rearing of *Corythucha ciliata*

Adults of *C. ciliata* were collected from London plane trees (*Platanus* × *acerifolia*) along Longwu Road, Shanghai, China at the beginning of May 2009. They were reared on leaves of *Platanus* × *acerifolia* in closed petri dishes (9 cm) with wet filter paper. The stock culture was conducted at a temperature of 26 ± 0.5° C, 80 ± 5% RH, and 14:10 L: D photoperiod in the laboratory. Progeny of laboratory-reared *C. ciliata* were used in these experiments. Fresh leaves from *P.* × *acerifolia* were supplied daily. Filter paper was changed at 2–3 day intervals and water was added onto paper to prevent desiccation.

### Effects of temperature on development and survivorship

A group of newly laid eggs (*n* ≥ 100) inside the leaf were placed in a closed petri dish (9 cm) with a piece of wet filter paper and then maintained at seven constant temperatures (16, 19, 22, 26, 30, 33, and 36 ± 0.5° C), at 80 ± 5% RH, and 14:10 L:D photoperiod in incubators (MIR 350H, Sanyo Electric Co., Ltd., www.sanyo.com). The eggs were observed daily and the wet filter paper was replaced where necessary. The total number of eggs hatching at each temperature was recorded and the duration of each egg development (incubation period) was recorded. After eggs hatched, a newly emerged nymph (*n* = 80) was put into a closed petri dish (9 cm) with a piece of leaf of *P.* × *acerifolia* and maintained under the same conditions through immature development. A visible exuvia was used as the evidence of molting when they were found among the frass of a developing nymph. Observations were undergone daily in order to measure survival and developmental time of each nymph until adults emerged. After adults finished eclosion, their sex ratio was obtained. If all eggs failed to hatch at a temperature, the first instar larvae reared at 26° C were supplied as experimental insects to determine the development and survival of immature stages at this temperature. The culture was maintained with fresh leaves as old materials deteriorated.

### Effects of temperature on fecundity, oviposition and longevity

One newly emerged female and one newly emerged male (less than 24 h old) were paired on the leaf into a closed petri dish (9 cm) with a wet filter paper. Fifteen pairs were tested at each treatment of six temperatures (16, 19, 22, 26, 30, and 33 ± 0.5° C). A new male was replaced whenever a male died. In this case, females had alternative males for mating during lifetime. Eggs together with the leaf were removed and the number of eggs laid was monitored daily. The number of eggs laid per female was counted at each temperature. The pre-oviposition period, oviposition period, and longevity of adults at each temperature were recorded. All the growing conditions (i.e. relative humidity, photoperiod, and fresh leaves as food source) were maintained as described above.

### Data analysis

Differences in developmental time, fecundity, longevity, pre-oviposition period, and oviposition period among temperature treatments were tested by one-way analysis of variance (ANOVA). If significant differences were detected, multiple comparisons were performed using Duncan's new multiple range test. Differences in longevity between female and male adults were analyzed by ANOVA and Student's-t test (SPSS 2006).

Developmental threshold temperature (Q and thermal constant (*K*) estimated for each stage were determined by following formulas (Chen et al. 2004):
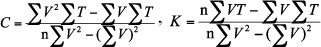

where *V* is the development rate at each temperature, *T* is the experimental temperature, and *n* is the number of temperature treatments.

Life table was constructed according to Morris-Watt model (Morris 1963), which is described as:
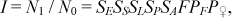

where *I* is population trend index; *N*1and *N*0are the numbers of next generation and current generation, respectively; *S_E_, S_S_, S_L_, S_P_* are the respective survival rates of eggs, lower-instar nymphs, higher-instar nymphs, and pupae; *S_A_* is the survival rate of adults; *F* the number of initial eggs at standard level (e.g. 100); *P_F_* is the number of average eggs laid by per female; and *P*♀ is the female proportion in adults.

## Results

### Effect of temperature on development and survivorship

**Figure 1.**
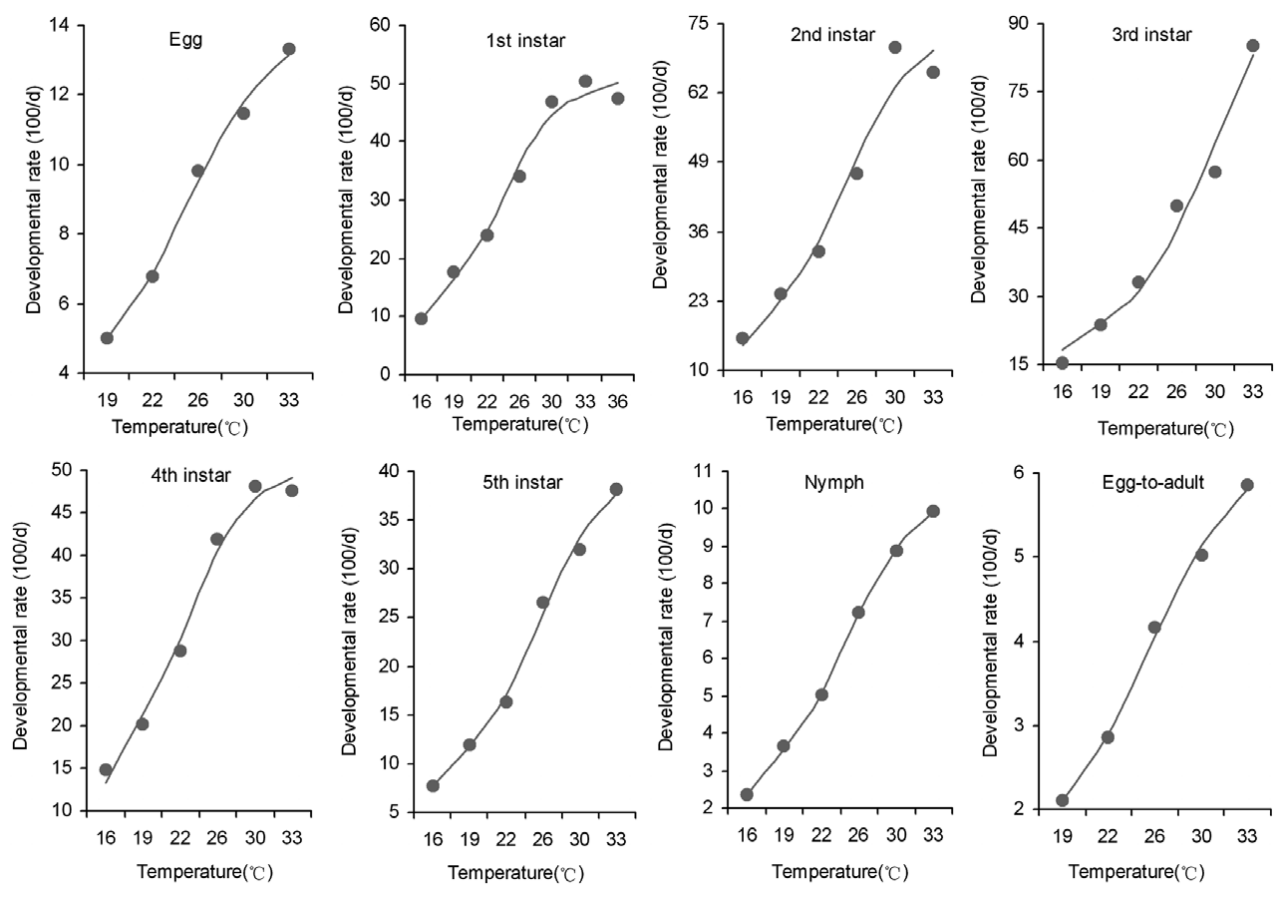
Developmental rate of *Corythucha ciliata* at different constant temperatures. Note that the curves are described by a logistic model. High quality figures are available online.

The developmental times for each stage of *C. ciliata* at seven constant temperatures are presented in Table 1. As expected, the average developmental time for each stage was significantly shortened as the temperature increased (egg: *F*4,461 = 5810.9, *P* < 0.0001; 1st instar: *F*6, 418 = 1675.1, *P* < 0.0001; 2nd instar: *F*5, _356_ = 265.0, *P* < 0.0001; 3rd instar: *F*5, 338 = 223.1, *P* < 0.0001; 4th instar: *F*5, 323 = 196.9, *P* < 0.0001; 5th instar: *F*5, _289_ = 465.1, *P* < 0.0001; total nymph: *F*5,289 = 1799.7, *P* < 0.0001; full development (from egg to adult): *F*4, 319 = 4147.1, P < 0.0001). The mean developmental time of eggs decreased from 20.0 days at 19° C to 7.6 days at 33° C, and that of nymphs decreased from 43.1 days at 16° C to 10.2 days at 33° C. No eggs hatched at 16° C and major nymphs did not develop at 36° C. Consequently, the overall duration from egg to adult was the longest (47.6 days) at 19° C and the shortest (17.1 days) at 33° C. Based on the data on the developmental time, the relationship between temperature and developmental rate was well described by a logistic model (Figure 1).

**Table 1.**
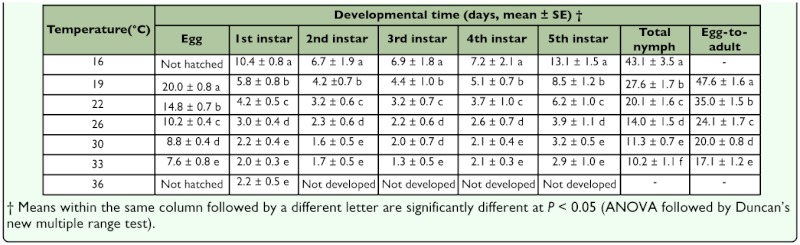
Developmental time for each stage of *Corythucha ciliata* at seven constant temperatures.

**Figure 2.**
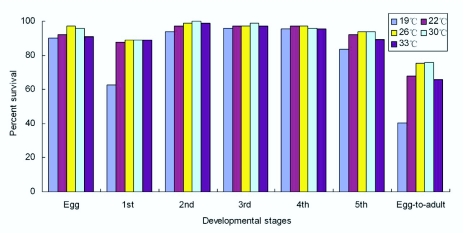
Survival of various stages of *Corythucha ciliata* at five constant temperatures. High quality figures are available online.

Survivorships of different stages of *C. ciliata* were significantly different at different temperatures (Figure 2). Of the developmental stages, survival rates of eggs and 2–4 instar nymphs were the highest (above 90%) at the five temperatures. Survival rate from egg to adult was the highest (75.60%) at 30° C and the lowest (40.50%) at 19° C (Figure 2).

### Developmental threshold temperature and thermal constant

**Table 2.**
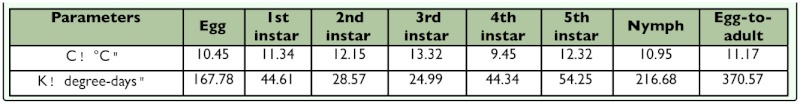
Developmental threshold temperature (C) and thermal constant (K) for each stage of *Corythucha ciliata.*

The developmental threshold temperature and thermal constant of *C. ciliata* differed among the developmental stages (Table 2). According to the development rates at each temperature, the estimated thermal constant (*K*) was 167.78 degree-days and threshold temperature (C) was 10.45° C for eggs, which were lower than those of nymphs and egg-to-adult. Among nymphs, the threshold temperature of the 4th instar was the lowest (9.45° C) and the 3rd instar was the highest (13.32° C). The threshold temperature and thermal constant from egg to adult were 11.17° C and 370.57 degree-days, respectively.

### Effect of temperature on oviposition, longevity and sex ratio

Mean fecundity of *C. ciliata* was significantly influenced by temperature (*F*4, _70_= 67.6, *P* < 0.0001) with the highest (286.8 eggs per female) being at 30° C and the lowest (87.7 eggs per female) at 19° C (Table 3). The females could mate with the males at 16° C, but failed to lay eggs. Most of the eggs were laid in groups or batches in the leaves around the main vein. Within the temperature range of 19–33° C, the relationship between the number of eggs laid and temperature followed a parabolic pattern: *y* = -2.13*x*2 + 122.82x 1492.40 (*r*2 = 0.94, *P* < 0.05); where *y* is the number of eggs laid, *x* is the temperature. From this equation, the temperature at which the maximum number of eggs was laid was estimated to be 28.47° C (when first-order derivative of the equation = 0).

Though the longevity of males was slightly higher than that of females at all temperatures, and no significant difference was detected between them at all temperatures (*t*28= 0.16–2.02, *P* > 0.05). Longevity of both females and males was significantly higher (female: *F*5, 84 = 14.7, *P* < 0.0001; male: F_5,__84_ = 12.4, *P* < 0.0001) at 16° C than that at other temperatures, with the shortest (female: 17.7 days; male: 19.7 days) being at 33° C (Table 3). Pre-oviposition and oviposition periods of the female adults significantly decreased (pre-oviposition: *F*4, 70 = 100.1, *P* < 0.0001; oviposition: *F*4, 70 = 60.8, *P* < 0.0001) with increasing temperature from 19–33° C (Table 3). At 30° and 33° C, females began to lay eggs within 4 days after molting to adults. However, the first lot of eggs was laid after 10.8 days at 19° C (Table 3). The oviposition period was the longest (16.8 days) at 19° C and the shortest (6.5 days) at 33° C. The sex ratio (female/male) of adults was the highest (2.86) at 16° C and the lowest (0.63) at 33° C.

**Table 3.**
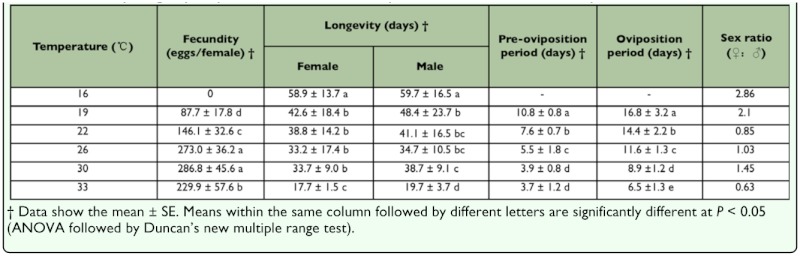
Fecundity, longevity, oviposition and sex ratio of *Corythucha ciliata* at six constant temperatures.

**Table 4.**
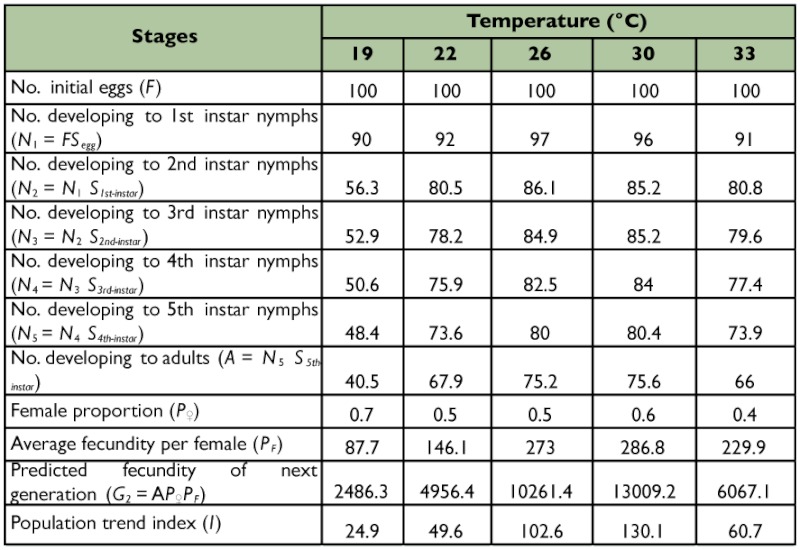
Life table of *Corythucha ciliata* population at five constant temperatures.

### Life table and population trend index

According to the data on survival rate, fecundity, and sex ratio, a life table of *C. ciliata* was constructed and is given in Table 4. In the table, standard eggs numbers (*F*) were set at 100, and hatching rate of eggs (*S_egg_*), survival rate of nymphs (*S_1st-instar_, S_2nd-instar_ S_3rd-instar_, S_4th-instar_* and *S_5th-instar_*) and female proportion (*P*♀) were based on the actual data in this study. Population trend index (*I*) measures the potential of population growth between next generation and current generation. From Table 4, the *I* value at 30° C was the highest among the five temperature treatments. The results indicated that the population increased 130.1 times after one generation at this temperature; that is, 30° C was the optimal temperature for *C. ciliata* population growth.

## Discussion

Temperature is an important factor, which exerts a profound influence on the development of insects. *C. ciliata* is not an exception. The effects of temperature on insect development may vary among species, but lower temperatures typically result in a decrease in the rate of development and increase in the duration of the time spent in each developmental stage. Results from this study indicated that the developmental time of different stages of *C. ciliata* declined with increasing temperature (Table 1). However, when the temperature was at 16° C eggs did not hatch, and when it was at 36° C neither eggs nor nymphs developed. The results obtained here were similar with those of Kim et al. (1999), who reported that *C. ciliata* could not complete normal development at 15° or 35° C. Thus, low and high temperatures had adverse effects on the development of *C. ciliata.* The temperature range of 19° to 33° C was suitable for the development of *C. ciliata* under laboratory conditions (Table 1). In fact, within the suitable temperature range for development of an insect, the relationship between temperature and development is often linear over the middle range of environmentally unconfined temperatures and becomes sigmoid across the entire temperature ranges through which insects are capable of development (Andrewartha and Birch 1954; Lactin et al. 1995; Arbab et al. 2006). In our study, the relationship between temperature and developmental rate for *C. ciliata* was better fit by logistic model for all stages at different temperatures (Figure 1).

Estimated threshold temperature and thermal constant for *C. ciliata* were similar to those reported in the literature. Kim et al. (1999) reported that threshold temperatures for egg, nymph, and whole generation of *C. ciliata* were respectively 11.10, 10.19, and 11.11° C and the thermal constants were respectively 150.13, 230.16, and 376.11 degree-days under laboratory conditions. Similarly, this study showed that threshold temperatures for egg, nymph, and egg-to-adult of *C. ciliata* were respectively 10.45, 10.95, and 11.17° C and thermal constants were respectively 167.78, 216.68, and 370.57 degree-days. Based on the data on thermal requirements obtained in the laboratory, and given the temperature in Shanghai, the population of *C. ciliata* is predicted to emerge from hibernating areas by about April when the average daily temperature is about 15° C. The prediction agrees with Ju et al. (2009), Xia et al. (2007), and Xiao et al. (2010) who reported that the first generation began to develop at the middle to end of April. Five generations of *C. ciliata* can be completed per year, but generally the fifth generation cannot be completed because daily temperature decreases below the developmental threshold. *C. ciliata* begins to hibernate at the end of October until the beginning of next April in Yangtze River basin in China (Xia et al. 2007; Xiao et al. 2010).

The current results indicated that adult longevity, pre-oviposition, and oviposition periods were also prolonged with decreasing temperature (Table 3). Fecundity of *C. ciliata* was higher at 26–30° C than that at other temperatures. Song and Cho (2000) reported that only 83 eggs were laid per female at 25° C, which was much lower than that at 26° C (273.0 eggs/per female) in this study (Table 3). This difference may be caused by the different host plants used in the two studies. d'Aguilar et al. (1977) counted 350 eggs laid per female in the wild, slightly higher than that obtained here in the laboratory. Riordan (1957) suggested that high temperatures might cause temporary or permanent sterility, or deactivation of the sperms stored in the spermatheca resulting in a reduced fertility. Our study demonstrated that females of *C. ciliata* failed to lay any eggs at 16° C, suggesting that low temperatures also induced sterility. Therefore, we suggest that both lower and higher temperatures would lead to developmental stagnancy of the ovaries.

Life tables are powerful tools for analyzing and understanding the impact that an external factor has upon the growth, survival, reproduction, and growth rate of an insect population (Bellows et al. 1992). In addition, the classical life table is primarily used to understand the age dynamics of adult populations studied under controlled laboratory conditions (Aysal and Kıvan 2008). Although many studies have examined the life history of *C. ciliata,* few life table studies have been conducted for the species in spite of its great value in pest management. Data from our study indicated that temperature had a strong effect on life table parameters through affecting the survival, longevity, and fecundity of *C. ciliata.* The population trend index (*I*) was the lowest at 19° C and the highest at 30° C (Table 4), indicating that the temperature of 30° C is the optimal for the growth and establishment of *C. ciliata* populations.

It is difficult, however, to know how well parameters estimated under the laboratory conditions at constant temperatures could be applied in the field (Omer et al. 1996) because under natural conditions, insects are never exposed to constant temperatures (Infante 2000). Nevertheless, laboratory studies at different temperatures can provide useful information on the development, survival, and reproduction of insects (Wang et al. 1997) that is essential to developing an effective IPM program. In such a program, chemical application is an important component for managing *C. ciliata* (Ju et al. 2009; Xiao et al. 2010). When applying this method some low-pollution insecticides such as Pyridines, Nicotine+Matrine, and EnSpray (a petroleum spray oil) are effective to control *C. ciliata* in urban areas (Ju et al. 2009). Timing for pesticide treatments can be improved if the experimentally determined thermal requirements are used to predict seasonal emergence of this species following hibernation as well as the regional probabilities of generation number. For example, in Shanghai the average daily temperature reaches the threshold temperature at the end of April, corresponding to the adult or nymph emergence. The prediction suggests that the optimal first insecticide application should be performed at this time, which may give the most effective control of *C. ciliata.*

In conclusion, temperature had significant effects on demography of *C. ciliata.* Results from this study showed that *C. ciliata* was sensitive to ‘extreme temperatures’ (e.g. 16° and 36° C) used here, with eggs failing to hatch and/or nymphs failing to complete the full development. Therefore, both low and high temperatures limited the survival and reproduction of *C. ciliata* and an optimal developmental temperature for the insect was 30° C. In the northern regions of China where *Platanus* trees are grown, the temperatures in summer often hover at around 30° C. Obviously, *C. ciliata* can easily get established in these regions. While in the southern or eastern China, the summer temperature can be greater than 36° C which may adversely influence the growth and development of *C. ciliata.* However, it is important to note that, the high temperature is not constant in nature and may have a fluctuation of about 10° C within a day. Therefore, whether high ambient summer temperatures in those regions limit the establishment of *C. ciliata* warrants further investigation.
